# Primary Care Consultations Among UK Police Officers and Staff: Links With Adverse Mental Health and Job Strain

**DOI:** 10.1097/JOM.0000000000002819

**Published:** 2023-02-26

**Authors:** Nora Trompeter, Nicola T. Fear, Neil Greenberg, Matthew Hotopf, Patricia Irizar, Simon Wessely, Sharon A.M. Stevelink

**Affiliations:** From the King's Centre for Military Health Research, Institute of Psychiatry, Psychology & Neuroscience, King's College London, United Kingdom (N.T., N.T.F., N.G., S.W., S.A.M.S.); Academic Department of Military Mental Health, King's College London, London, United Kingdom (N.T.F.); Department of Psychological Medicine, Institute of Psychiatry, Psychology & Neuroscience, King's College London, London, United Kingdom (M.H.); and Department of Sociology, School of Social Sciences, University of Manchester, Manchester, United Kingdom (P.I.).

**Keywords:** police, help seeking, primary care, mental health, job strain

## Abstract

The current study examined links between adverse mental health, job strain, and likelihood and frequency of primary care consultations among police employees.

**Methods:**

We conducted secondary data analysis on the Airwave Health Monitoring Study data (*n* = 33,730). Measures included self-report data on mental health, job strain, job support, and primary care consultations in the past 12 months. Data were analyzed using a zero-inflated Poisson regression framework.

**Results:**

Findings showed that overall, help seeking was low based on mental health status and job strain. Adverse mental health was associated with more primary care consultations. Police employees with high, active, or passive job strain reported more primary care consultation compared with police employees with low strain.

**Conclusions:**

Findings suggest that more work to reduce stigma of taking the initial step of help seeking would be beneficial.

## Abstract 2

This work reveals a potential unmet need among police employees with adverse mental health and high job strain in primary care. There may be opportunities for the police force to further reduce the stigma of taking the initial step of help-seeking.

**LEARNING OUTCOMES**

- After reviewing this article, readers should be able to identify the links between job strain, adverse mental health, and primary care consultations.
- After reviewing this article, readers should be able to describe the impact of job support on help seeking among police employees.

Policing has consistently been linked with poor physical and mental health.^1^ Evidence has reported increased mental health problems among police employees, which is often attributed to occupational stressors.^2^ Police officers frequently rate occupational stressors, such as job strain, as more stressful than exposure to potentially traumatic events, such as dealing with physical aggression.^3^ Despite high rates of adverse mental health and occupational stressors, few police employees seek professional help.^4^ Indeed, symptoms of posttraumatic stress disorder (PTSD) have been linked to a lower likelihood of organizational support service utilization, such as workplace counseling, among police officers.^5^ However, little is known about how likely police employees are to utilize non-organizational health services, such as state-funded primary care health services. Understanding how likely police employees, at risk of adverse mental health, are to use primary care health services would allow employers to better cater for their health needs. The current study looked at whether adverse mental health and high job strain are linked to both likelihood and frequency of primary care consultations among UK police employees.

The job demand-control model proposes that job strain depends on two key factors: job demands and job control.^6^ Job demands include aspects such as workload, time pressure, and physical and emotional demands. Job control refers to the extent individuals are capable of controlling their tasks and general work activity. According to the model, individuals in a high strain job (high demands and low control) are likely to report the lowest well-being.^6^ This model fits well with the results of a recent systematic review that identified job demand as being consistently linked with adverse mental health.^7^ Later iterations of the job demand-control model include perceived job support as a potential protective factor that might prevent negative outcomes otherwise associated with high job strain.^8^ Perceived job support includes aspects of social support at work (eg, by colleagues or supervisors) and a supportive work environment (eg, spirit of unity among employees). Indeed, recent guidelines from the World Health Organization highlight the need to provide job support to effectively manage workers' mental health.^9^ So far, evidence for the protective effect of job support has been mixed with a systematic review of the model finding that only 4 of 11 studies were supporting the protective effect of job support on general psychological well-being.^10^

Findings among police employees have also been mixed. A recent study among Polish police officers supports the model, with high job support attenuating the association between high job demand and depressive symptoms.^11^ Similarly, among Norwegian police officers, both high job demand and low job support were associated with adverse mental health and burnout.^12^ In contrast, previous research among UK police employees from more than 40,000 police employees in the Airwave study found no moderating effect of job support on the relationship between job strain and alcohol use or binge drinking.^13^ Thus, more research on the specific role of job strain on mental health status among police employees is needed. Furthermore, it remains unknown whether police employees who experience high job strain are likely to seek professional help and whether job support increases such help seeking. Conversely, it is also possible that high levels of job support may reduce the need for employees to seek help elsewhere.

Although previous research on help seeking by police employees has primarily focused on services offered by organizations,^14^ little information on the uptake of primary care consultations is available. This is important, as previous research indicates that police employees with mental health problems are more likely to seek help outside of their organizational support systems, in particular for those with problems relating to alcohol use and PTSD.^15^ Primary care physicians are often the first point of contact for individuals with mental health difficulties or those experiencing high levels of distress.^16^ Thus, understanding whether police employees with adverse mental health or high job strain (ie, those likely in need of medical support) access primary care consultations and at what frequency is crucial in building better support systems. Despite provisions of organizational support services (eg, workplace counseling), for the vast majority of workers, primary care services are the mainstay of health care, even when related to occupational matters.^17^ Effective organizational management of employee well-being is therefore reliant on both the provision of care and promotion of help seeking more broadly, and requires knowledge about help seeking within primary care.

To address these gaps, the current study aimed to examine the link between adverse mental health, job strain, and the likelihood and frequency of primary care consultations among police employees. Furthermore, an interaction with job support was considered. To investigate the overlap between adverse mental health and job strain, we tested a potential interaction between these factors. Lastly, an exploratory analysis was conducted among “at-risk” police employees (ie, probable mental disorder or high job strain) to examine which sociodemographic factors (eg, age or sex) were associated with primary care consultations.

## METHODS

### Study Sample

This study uses the Airwave Health Monitoring Study data.^18^ In brief, the Airwave study examined potential health impacts of the Terrestrial Trunked Radio, a digital communication system used by emergency services since 2001. As part of the study, police employees completed a self-report questionnaire and a health screen conducted by trained nurses. Data collection took place between June 2006 and March 2015. In total, 53,114 police employees participated from 28 participating forces across the United Kingdom, of whom 45,567 (85.8%) completed the self-report questionnaire and 45,514 (85.7%) completed the health screen. Although no information on attrition is available, within most forces, both the percentage of men and the average age of participants were similar to those of the overall force.^18^ For this analysis, participants who indicated their role (ie, police officer or police staff) as “other” (*n* = 738) or did not have data available on their role (*n* = 8245) were excluded because of limited confidence that responses were that of police employees. To minimize data reduction, we backfilled data on rank from employment data available through the Airwave study, which resulted in 94 completed cases. Data were used from all police employees who had answered the question regarding primary health care consultations as part of the self-report questionnaire (*n* = 33,730 [63.5% of the total sample]). Ethical approval for The Airwave Health Monitoring Study was granted by the National Health Service multisite research ethics committee (MREC/13/NW/0588). All participants provided written informed consent before participating.

### Measures

#### Primary Care Consultations

Participants were asked a single-item question on primary health care consultations in the previous year (“In the past year how many times have you consulted your GP for your health problems?”). Participants responded using a drop-down menu ranging from 0 to 20+, with each interval representing one consultation. Responses were treated as a count variable representing the number of primary care consultations in the previous year.

#### Adverse Mental Health

To examine adverse mental health, we used data on probable depression (Patient Health Questionnaire [PHQ-9]),^19^ probable anxiety disorder (Hospital Anxiety and Depression Scale—Anxiety [HADS-A]),^20^ and probable PTSD (Trauma Screening Questionnaire [TSQ]),^21^ The PHQ-9 asked participants to indicate how frequently they had experienced nine different symptoms of depression over the previous 2 weeks on a 4-point Likert scale (0 [not at all] to 3 [nearly every day]). Probable depression was assigned using the validated cutoff score of 10.^19^ Similarly, the HADS-A asked participants to rate the frequency of seven different symptoms of anxiety over the past 2 weeks on a 4-point Likert scale (0 [not at all] to 3 [most of the time/very much indeed]). Probable anxiety was assigned using the validated cutoff score of 11.^22^ For the TSQ, participants were first asked whether they had been bothered by a disturbing incident that had occurred in the past 6 months. If they endorsed this item, participants were asked to indicate whether 10 different PTSD symptoms applied to them since that incident. Unlike the original TSQ measure, which used a yes/no response format, the Airwave Health Monitoring Survey asked participants to rate symptoms on a 5-point Likert scale (0 [not at all] to 4 [extremely]). Thus, in line with previous publications,^23^ all responses 1 to 4 were coded as 1. Probable PTSD was assigned using the validated cutoff score of 6.^21^ All scales showed acceptable internal consistency (PHQ-9, *α* = 0.85; HADS-A, *α* = 0.67; TSQ, *α* = 0.92).

#### Job Demand, Control, and Support

As part of the Airwave Health Monitoring Survey, 10 items from the Job Content Questionnaire^24^ were presented: 2 items on job demands, 4 items on job control, and 4 items on support. Participants rated how much they agreed with each item on a 4-point Likert scale (1 [strongly agree/often] to 4 [strongly disagree/never]). Based on the original scale model, participants were categorized into four distinct categories of job strain using a quadrant approach based on the sample median: *high strain* (high demands, low control), *low strain* (low demands, high control), *active strain* (high demands, high control), and *passive strain* (low demands, low control). Job support was included as a separate continuous variable, with scores ranging from 4 to 16 whereby higher scores indicate higher perceived job support. All subscales demonstrated acceptable internal consistency (job demand, *α* = 0.73; job control, *α* = 0.67; job support, *α* = 0.83).

### Control Variables

#### Sociodemographic

To control for sociodemographic influences on primary care consultations, we included the following factors as control variables: rank (police officer or police staff), years in current role, salary, educational attainment, marital status, ethnicity, age, and sex.

#### Job Satisfaction

Participants were asked to rate their job satisfaction on a single item using a 4-point Likert scale (1 [very dissatisfied] to 4 [very satisfied]). Scores on this variable were highly skewed, with most participants rating their job satisfaction as either satisfied or very satisfied (82.29%), and assumption of a normal distribution was violated (Shapiro-Wilk test: *z* = 11.74, *P* < 0.001). As such, this variable was treated as a categorical variable with two levels: very dissatisfied/dissatisfied and very satisfied/satisfied.

#### Physical Health

To control for participant's physical health, we included the following factors as control variables: self-reported health condition (yes/no on any health condition excluding depression) and diastolic blood pressure (taken by a nurse at a separate health assessment). In the list of health conditions, depression was the only listed mental health problem. Because the purpose of controlling for physical health indicators was to minimize the influence of physical health factors on help seeking, we did not include self-reported depression in this variable. Because some participants presented to the health assessment multiple times, we excluded data on blood pressure from these participants, as it was unclear which visit corresponded with their survey data. In line with NHS guidelines, we categorized diastolic blood pressure into three categories: low diastolic blood pressure (<60 mm Hg), normal diastolic blood pressure (60–89 mm Hg), and high diastolic blood pressure (90 mm Hg or higher).

### Data Analysis Plan

The data analysis plan was preregistered (https://osf.io/awgme/?view_only=e4d0c2bad44a4496b3bd88ab2a188ced). All deviations from this plan are explicitly mentioned hereinafter. As planned, analyses were conducted in a zero-inflated Poisson regression framework in Stata version 16 (StataCorp LLC, College Station, TX).^25^ The zero-inflated Poisson regression uses a mixture of a Poisson distribution of count data (eg, number of primary care consultations) with an excess of zero counts. Using this regression, the occurrence of the outcome (zero-inflated part) and the frequency of the outcome (Poisson part) were examined separately in the same model.

To test the study aims, we conducted four separate regression analyses. The main outcome of interest was twofold: (1) likelihood of at least one past-year primary care consultations and (2) frequency of past-year primary care consultations. The first model examined the link between adverse mental health and primary care consultation likelihood and frequency, the second model examined the link between job strain and primary care consultations, and finally, the third model included both factors (adverse mental health and job strain). In the final model, we also tested the interaction between these factors. For simplicity, we used a combined “any mental health problem” to capture individuals who met the criteria for a probable anxiety disorder, depression, or PTSD in the final model. The first two models additionally considered an interaction with job support. We had originally planned to include binge drinking as another facet, alongside adverse mental health and job strain. However, these analyses will be reported in a future publication instead.

All analyses controlled for sociodemographic factors and physical health factors that had a significant association with primary care consultations in univariate analyses. To deal with missing data, we first examine missing data patterns to determine whether data were missing at random. In total, there were 8% of missing data. Missing data were significantly associated with sex, job rank, salary, age, and education. To account for these associations, these factors were controlled for in all analyses, even if no significant associations with primary care consultations emerged in univariate analyses. Although we had initially planned to use multiple imputation to deal with missing data, this was not deemed appropriate given that the data were not missing at random.^26^ Instead, we used list-wise deletion to deal with missing data.^27,28^

For exploratory analyses, we examined sociodemographic factors linked with primary care consultations for individuals who met the criteria for “any mental health problem” or were classified as experiencing “high job strain.” Analyses were carried out in a zero-inflated Poisson regression. Physical health variables were included as covariates.

## RESULTS

### Sample Characteristics

The sample characteristics (*n* = 33,730) are described in Table 1. Both the ethnicity and sex composition of the sample are representative of the broader police force, where 92.4% of police officers are White and 67.6% are male.^29^

**TABLE 1:** Univariate Regression Analysis Examining the Likelihood and Frequency of Primary Care Consultations in the Past 12 Months by Sociodemographic and Physical Health Factors (*n* = 33,730)

	Primary Care Consultations								
	Likelihood					Frequency			
	*n*	%	OR	*P*	95% CI	IRR	*P*	95% CI
Age, yr								
<30	4556	13.5	Reference					
30–39	11,043	32.7	0.91	0.122	0.80–1.03	1.00	0.860	0.97–1.03
40–49	13,301	39.4	**0.85**	**0.009**	**0.76–0.96**	0.97	0.084	0.94–1.01
≥50	4830	14.3	1.04	0.596	0.90–1.20	**1.11**	**<0.001**	**1.07–1.15**
Sex								
Female	12,416	36.8	Reference					
Male	21,310	63.2	**0.63**	**<0.001**	**0.58–0.68**	**0.69**	**<0.001**	**0.68–0.71**
Ethnicity								
White	1632	4.9	Reference					
Non-White	31,926	95.1	**1.21**	**0.030**	**1.02–1.43**	**1.16**	**<0.001**	**1.11–1.21**	
Education									
Low (O levels/GCSEs or none)	11,249	33.5	Reference						
High (A levels, degree or higher)	22,639	66.5	1.03	0.474	0.95–1.11	**0.94**	**<0.001**	**0.93–0.97**	
Marital status									
Married/cohabiting	26,162	77.8	Reference						
Divorced/separated	2703	8.0	1.14	0.065	0.99–0.1.30	**1.16**	**<0.001**	**1.12–1.20**	
Single	4023	12.0	0.89	0.059	0.82–1.00	**1.15**	**<0.001**	**1.11–1.18**	
Other	730	2.2	1.22	0.128	0.94–1.57	**1.18**	**<0.001**	**1.11–1.25**	
Rank								
Police officer	23,951	71.0	Reference					
Police staff	9779	29.0	**1.32**	**<0.001**	**1.22–1.43**	**1.26**	**<0.001**	**1.23–1.28**
Years in current role								
≤5 yr	22,126	65.6	Reference					
6–10 yr	6942	20.6	0.99	0.812	0.90–1.09	1.02	0.067	1.00–1.05
11–20 yr	3527	10.5	**0.87**	**0.011**	**0.77–0.97**	**1.06**	**<0.001**	**1.03–1.10**
≥20 yr	1135	3.4	1.03	0.813	0.83–1.26	1.01	0.619	0.96–1.07
Salary									
≤£25,999	7480	22.3	Reference						
£26,000–£37,999	14,006	41.7	**0.85**	**0.001**	**0.77–0.93**	**0.85**	**<0.001**	**0.83–0.87**	
£38,000–£59,999	11,095	33.0	**0.80**	**0.001**	**0.72–0.89**	**0.71**	**<0.001**	**0.69–0.73**	
≥£60,000	1037	3.1	**0.69**	**<0.001**	**0.54–0.89**	**0.63**	**<0.001**	**0.58–0.68**	
Total working hours (excluding overtime)									
≤40 h/wk	8444	25.5	Reference						
41–48 h/wk	20,971	63.3	**0.75**	**<0.001**	**0.69–0.82**	**0.78**	**<0.001**	**0.77–0.80**	
≥49 h/wk	3737	11.3	**0.74**	**<0.001**	**0.65–0.85**	**0.77**	**<0.001**	**0.74–0.80**	
Job satisfaction									
Dissatisfied/very dissatisfied	5777	17.4	Reference						
Satisfied/very Satisfied	27,104	82.6	**0.86**	**0.002**	**0.78–0.95**	**0.83**	**<0.001**	**0.81–0.85**	
Blood pressure									
Low	541	1.7	**1.58**	**0.023**	**1.07–2.37**	0.95	0.247	0.88–1.03	
Normal	26,279	83.8	Reference						
High	4556	14.5	0.97	0.548	0.87–1.08	**1.05**	**0.001**	**1.02–1.08**	
Medical diagnosis (excluding depression)									
None	13,027	38.6	Reference						
Any	20,703	61.4	**1.58**	**<0.001**	**1.45–1.71**	**1.45**	**<0.001**	**1.42–1.48**	

Significant associations are bolded.

CI, confidence interval; GCSE, General Certificate of Secondary Education; IRR, incidence rate ratio; OR, odds ratio.

In total, 38.6% of the sample reported at least 1 health condition out of a list of 20 conditions, excluding mental health problems. Of these, the most common health conditions were allergies (*n* = 5187 [39.8%]), asthma (*n* = 3408 [26.2%]), and migraine (*n* = 2536 [19.5%]). Most participants with a health condition reported having one (70.7%) or two conditions (23.3%). In terms of primary care consultations, most people reported having at least one primary care consultation in the past 12 months (*n* = 23,342 [69.2%]), with a mean (SD) of 1.7 (2.1) consultations (median, 1; interquartile range, 0–2). Primary care consultations in the current sample were substantially lower compared with the average UK population (ie, including nonworking individuals), with an average of 3.8 primary care consultations over 12 months.^30^

### Univariable Associations

Table 1 shows findings from the univariate analyses examining the association between sociodemographic and occupational characteristics, and physical health with both the likelihood of attending a primary care consultation and the frequency of primary care consultations in the previous 12 months. Police employees were more likely to attend a primary care consultation within the past 12 month if they were younger than 30 years, female, non-White ethnicity, police staff, and in their current role for less than 5 years; had a lower salary; worked fewer hours; had low blood pressure; and reported a medical condition. Police employees attended more primary care consultations in the past 12 months if they were older than 50 years, were female, were non-White ethnicity, did not complete their A levels, were not married or cohabitating, were police staff, were in their current role for 11 to 20 years, had a lower salary, worked fewer hours, had high blood pressure, and reported a medical condition.

Based on these findings, all sociodemographic, occupational, and physical health variables were included as covariates in the subsequent multivariate analyses.

### Main Analyses

#### Adverse Mental Health

A total of 6074 participants (19.2%) reported meeting the criteria for a mental health problem. In total, 9.8% met the criteria for probable depression, 12.2% for probable anxiety, and 4.0% for probable PTSD. Findings showed that only police employees with a probable anxiety disorder were more likely to have attended a primary care consultation in the past 12 months, compared with police employees without an anxiety disorder (Table 2). However, police employees with probable depression, anxiety, PTSD, or a combination of the three had more primary health consultations in the past 12 months compared with police employees without mental health problems. Notably, this association was stronger for probable depression compared with probable anxiety disorder or probable PTSD.

**TABLE 2:** Adjusted Regression Analysis Examining the Likelihood and Frequency of Primary Care Consultations in the Past 12 Months by Adverse Mental Health Status (Step 1) and Interaction With Job Support (Step 2; *n* = 33,730)

		Likelihood			Frequency			
		AOR	*P*	95% CI	AIRR	*P*	95% CI
Step 1	Probable depression	1.15	0.089	0.98–1.36	**1.42**	**<0.001**	**1.37–1.46**
	Probable anxiety disorder	**1.30**	**0.001**	**1.11–1.52**	**1.23**	**<0.001**	**1.19–1.26**
	Probable PTSD	1.04	0.762	0.83–1.30	**1.16**	**<0.001**	**1.11–1.22**	
	Job support	0.99	0.280	0.97–1.01	**1.01**	**0.018**	**1.01–1.01**	
Step 2	Probable depression × job support	1.05	0.113	0.99–1.10	1.01	0.074	0.99–1.02	
	Probable anxiety disorder × job support	1.03	0.287	0.98–1.09	0.99	0.906	0.99–1.01	
	Probable PTSD × job support	1.00	0.907	0.91–1.08	**1.03**	**<0.001**	**1.01–1.04**	

Significant associations are bolded. Reference category was “no case” for the adverse mental health variables. Analysis adjusted for sex, age, ethnicity, education, rank, years in role, salary, working hours, job satisfaction, blood pressure, and medical diagnosis.

AIRR, adjusted incidence rate ratio; AOR, adjusted odds ratio; CI, confidence interval; PTSD, posttraumatic stress disorder.

**FIGURE 1:**
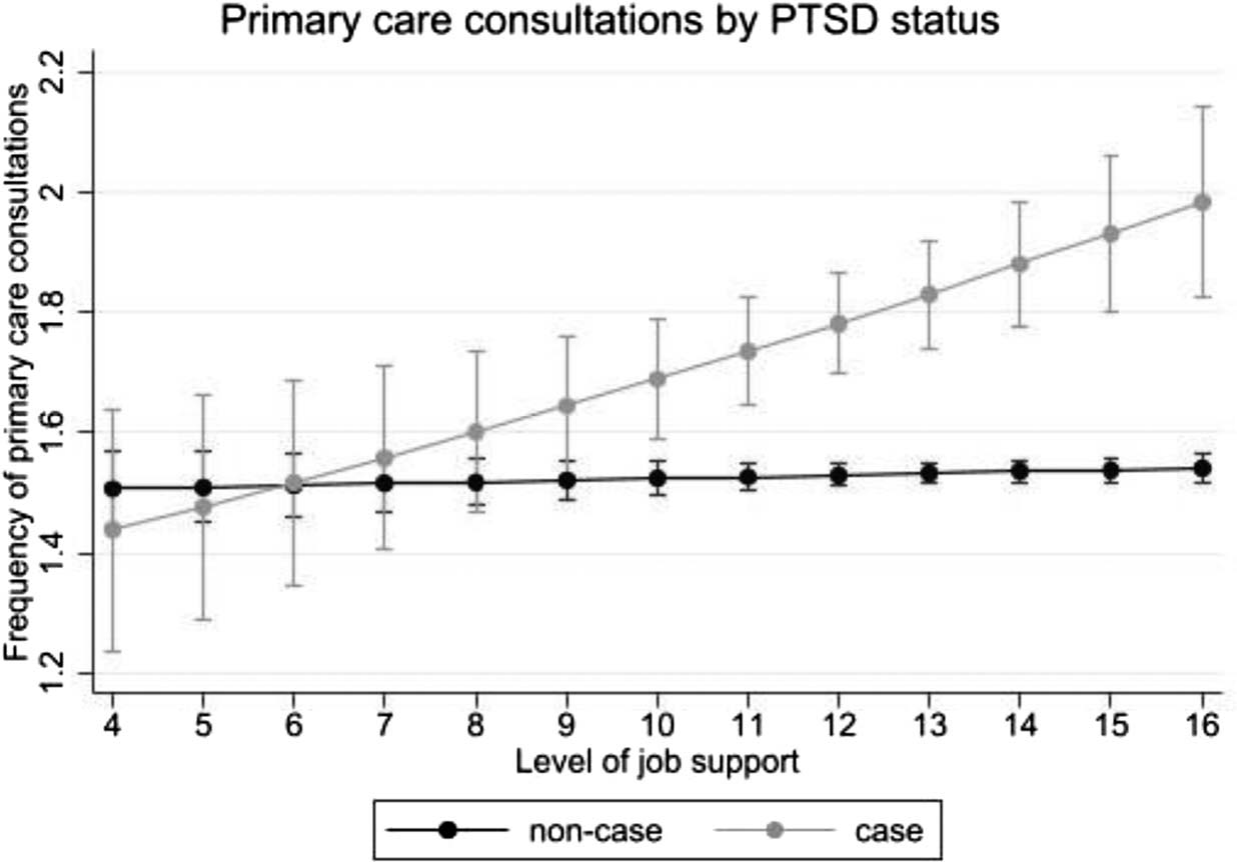
Interaction between job support and probable PTSD regarding their association with frequency of primary care consultations. PTSD, posttraumatic stress disorder.

In addition, there was an interaction between job support and probable PTSD, but not probable depression or anxiety, in the association with frequency of primary care consultations. As can be seen in Figure 1, higher levels of job support were associated with more primary care consultations among police employees with probable PTSD, but not among those without probable PTSD.

#### Job Strain

Around 27.7% of participants were categorized as having low job strain, 23.7% as high job strain, 28.3% as active job strain, and 20.3% were categorized as having passive job strain. Police employees with high strain, active strain, or passive strain attended more primary care consultations than police employees with low strain (Table 3). This effect was most pronounced for those in the high strain category. Job support was not associated with likelihood or frequency of primary care consultations, nor did it interact with job strain.

**TABLE 3:** Adjusted Regression Analysis Examining the Likelihood and Frequency of Primary Care Consultations in the Past 12 Months by Job Strain (Step 1) and Interaction With Job Support (Step 2; *n* = 33,730)

		Likelihood			Frequency			
		AOR	*P*	95% CI	AIRR	*P*	95% CI
Step 1	High strain vs low strain	0.95	0.459	0.83–1.09	**1.11**	**<0.001**	**1.07–1.15**
	Active strain vs low strain	0.98	0.798	0.86–1.12	**1.07**	**<0.001**	**1.04–1.10**
	Passive strain vs low strain	1.00	0.958	0.87–1.01	**1.04**	**0.030**	**1.01–1.07**	
	Job support	0.99	0.182	0.96–1.01	1.00	0.614	0.99–1.00	
Step 2	High strain × job support	0.95	0.095	0.90–1.01	0.99	0.407	0.98–1.01	
	Active strain × job support	0.97	0.332	0.92–1.03	1.00	0.903	0.98–1.01	
	Passive strain × job support	0.96	0.147	0.90–1.02	1.00	0.501	0.99–1.02	

Significant associations are bolded. Analysis adjusted for sex, age, ethnicity, education, rank, years in role, salary, working hours, job satisfaction, blood pressure, and medical diagnosis.

AIRR, adjusted incidence rate ratio; AOR, adjusted odds ratio; CI, confidence interval.

#### Combined Factors

When examining adverse mental health and job strain together, findings remained largely unchanged (Table 4). One exception was passive job strain, which was no longer associated with frequency of primary care consultation when compared with low strain.

**TABLE 4:** Adjusted Regression Analysis Examining Likelihood and Frequency of Primary Care Consultations in the Past 12 Months by Adverse Mental Health and Job Strain (*n* = 33,730)

		Likelihood			Frequency			
		AOR	*P*	95% CI	AIRR	*P*	95% CI
Step 1	Probable mental health problem	**1.22**	**0.001**	**1.08–1.37**	**1.48**	**<0.001**	**1.45–1.52**
	High strain vs low strain	0.96	0.603	0.84–1.11	**1.06**	**<0.001**	**1.03–1.09**
	Active strain vs low strain	1.01	0.947	0.88–1.15	**1.03**	**0.036**	**1.00–1.06**
	Passive strain vs low strain	1.05	0.494	0.91–1.22	1.02	0.349	0.98–1.05	
Step 2	Mental health × high strain	1.03	0.874	0.75–1.41	**0.91**	**0.005**	**0.85–0.97**	
	Mental health × active strain	1.06	0.714	0.76–1.48	0.94	0.086	0.88–1.01	
	Mental health × passive strain	**1.48**	**0.044**	**1.01–2.18**	0.95	0.113	0.88–1.01	

Significant associations are bolded. Analysis adjusted for sex, age, ethnicity, education, rank, years in role, salary, working hours, job satisfaction, blood pressure, and medical diagnosis.

AIRR, adjusted incidence rate ratio; AOR, adjusted odds ratio; CI, confidence interval.

Furthermore, there was a significant interaction between adverse mental health and high job strain. Specifically, among police employees with a probable mental health problem, there was no difference in primary care consultations between police employees experiencing high job strain compared with those experiencing low job strain. In contrast, among police employees without a mental health problem, those with high job strain had more primary care consultations compared with those with low job strain. In addition, there was an interaction between adverse mental health and passive job strain. That is, police employees with a probable mental health problem with passive job strain were more likely to attend a primary care consultation compared with those without a mental health problem and low job strain.

#### Exploratory Analysis

As exploratory analyses, we examined the sociodemographic factors linked with primary care consultations among police officers with a probable mental health problem or those experiencing high job strain (*n* = 11,461 [34.3%])—that is, those deemed “at risk” for psychological problems requiring formal health care (Table S1, http://links.lww.com/JOM/B304). Few significant associations emerged when examining the likelihood of primary care consultations. Police employees deemed to be “high risk” were more likely to attend a primary care consultation if they were female, married, or cohabitating as opposed to single, police staff, or in their current role for less than 5 years. Several factors were linked to frequency of primary care consultations. Police employees deemed to be “high risk” had attended more primary care consultations if they were older, were female, were non-White, were not married, had a lower salary, and had fewer working hours. The only factor associated with both reduced likelihood and frequency of primary care consultations was being male, which also had the biggest effect size. Conversely, being single compared with married/cohabitating was associated with lower likelihood of attending a primary care consultation, but higher frequency.

## DISCUSSION

The current study aimed to examine the link between adverse mental health, job strain, and the likelihood and frequency of primary care consultations among UK police employees. Findings showed that police employees with a probable anxiety disorder were more likely to attend a primary care consultation, compared with those without a probable anxiety disorder. In addition, adverse mental health was associated with frequency of primary care consultations, particularly for those with probable depression. Neither probable depression nor probable PTSD had a unique association with higher likelihood of primary care consultations. Thus, there may be a difference between different types of adverse mental health and likelihood of attending primary care consultations that should be investigated further. Overall, adverse mental health reported by police employees was similar to that in the general working population^31^ and similar occupation groups like the military.^32,33^

When examining job strain, findings were in line with the job demand-control model,^6^ whereby effects were most pronounced for police employees reporting high job strain. That is, police employees with high job strain, active job strain, and passive job strain reported more frequent primary care consultations compared with police employees with low strain. There was no difference in likelihood of primary care consultations. Consequently, police employees with higher job strain may not necessarily recognize the need to seek help but do require more medical attention. This is in line with previous research among the general working population, where links between job strain and sick days have frequently been reported.^34,35^ Thus, this study provides further evidence for the link between job strain and health problems. However, job support seemed to have no notable links with primary care consultations in the current study. Interestingly, there was a significant interaction between job support and probable PTSD in that higher job support was associated with more primary care consultations among police employees with probable PTSD, but not those without. These findings suggest that, although job support has little influence on help seeking among police employees, job support can be beneficial for those most in need of additional help. Furthermore, because police work is inherently linked to potentially traumatic exposures, PTSD may be more recognized by managers and spoken about in a work setting, which may foster a supportive environment that facilitates help seeking.

Furthermore, the current study found a significant interaction between adverse mental health and job strain. That is, among police employees without a mental health problem, those with high job strain had more primary care consultations compared with those with low job strain. In contrast, among police employees who reported a probable mental health problem, those experiencing high job strain did not differ in primary care consultations compared with those experiencing low job strain. This suggests that, although police employees without a probable mental health problem might be more likely to notice the additional support needed to handle the impact of experiencing high job strain, those also experiencing adverse mental health may be less likely to recognize the additional impact of high job strain on their mental health. Indeed, previous research has found that one of the mechanisms linking high job strain with adverse mental health is a lack of detachment (ie, thinking about work when at home).^36^ Police employees with adverse mental health may already experience problems with detachment, regardless of job strain, unlike police employees without adverse mental health for whom the impact of high job strain may be more noticeable.

Findings from our exploratory analyses found few structural factors related to likelihood of primary care consultations among those “at risk.” A notable finding was the strong association between sex and both likelihood and frequency of primary care consultations, with men significantly less likely to attend a primary care consultation and attending fewer consultations compared with women. Although this is in line with more general sex differences in mental health help seeking,^37^ it provides an important avenue for the police to actively encourage male employees to seek help and tackle both stigma toward mental health in general, but also gender-based stigma.

The current study has several implications for organizational support within the police force and similar organizational structures. First, the current study suggests that police employees who might benefit from formal medical support (ie, those with adverse mental health, or high job strain) report the same likelihood of attending primary care consultations compared with their peers. However, there was evidence that police employees with adverse mental health or high job strain attended more primary care consultations. Thus, efforts should focus on encouraging police employees to take the initial step of seeing their primary care physician, as current findings suggest that once police employees have engaged with primary care, they will continue with treatment as needed. Stigma is a frequently reported barrier for police employees seeking help for mental health problems,^5^ which many police employees experience as a key part of police culture.^38^ This may be addressed through organizational efforts, although it is important to note that stigma is a societal issue not restricted to the police force.^39^ Indeed, a recent study among Canadian police employees found that having completed mental health training (ie, Mental Health First Aid course) was positively associated with help seeking.^40^ In addition, the current study highlights the impact of job strain on mental health, with police employees experiencing high, active, or passive job strain reporting significantly more primary care consultations compared with police employees with low job strain. Although it is encouraging to see this level of help seeking, it further reinforces the adverse impact of low job control and high job demands. Thus, police forces should strive to further reduce these factors to promote well-being among employees.

Although the current study had many strengths, including the large representative sample, several limitations should be noted. First, the study relied purely on self-report data. In particular, reporting on primary care consultations in the previous 12 months may have been subject to recall bias with police employees potentially underestimating or overestimating the number of consultations they attended. Specifically, recall bias may differ between individuals with a probable mental health problem and those without a mental health problem, whereby individuals with mental health problems frequently overestimate negative events and underestimate positive events.^41^ Second, the data were collected over several years during which several changes in health care were implemented. For example, in 2008 the United Kingdom introduced an alternative pathway to accessing low-intensity mental health treatment (Improving Access to Psychological Therapies), which people can access without a referral from their primary care physician^42^ and is commonly used for treating low-level depression and anxiety. Thus, police employees may have visited their primary care physician less but attended Improving Access to Psychological Therapies sessions instead. However, no information on this was available in the current study. Similarly, the study did not examine the reasons for a primary care consultation, and we do not know whether police employees were attending consultations to seek help for their mental health. Although we did control for some aspects of physical health (eg, blood pressure and history of a medical condition), future research should examine mental health help seeking more explicitly. In addition, primary care consultations referred to the 12 past months only, and we did not have historical data available. It is therefore unknown to what degree participants accessed primary care consultations in the preceding years. Lastly, the current study relied on cross-sectional data, and no inferences about causality can be made. Future research should examine whether experiences of adverse mental health and high job strain lead to subsequent primary care consultations.

In conclusion, the current study showed that police employees with adverse mental health and high job strain reported more primary care consultations in the previous 12 months compared with police employees without adverse mental health and low job strain. Although some police employees with adverse mental health were also more likely to have had any primary care consultations, this association was not observed for police employees with high job strain. These results suggest that more work to reduce stigma of taking the initial step of help seeking would be beneficial. This is especially true for male police staff who were the least likely to seek help from primary care. Furthermore, although it is laudable that high job support was linked with more frequent primary care consultations among police employees with probable PTSD, it would be useful to help police managers better understand the potential benefits of staff with anxiety disorders or depression also seeking help, especially if their symptoms are severe and impairing occupational function.

## Supplementary Material
