# Supplementary material for: Primary Care Consultations Among UK Police Officers and Staff: Links With Adverse Mental Health and Job Strain
Source: J Occup Environ Med. 2023 Feb 26;65(6):502–9. doi: 10.1097/JOM.0000000000002819 (PMC10227927; doi:10.1097/JOM.0000000000002819)
Supplement: Supplementary file 1 [file joem-65-0502-s001.docx]

**Primary care consultations among UK Police officers and staff: Links with adverse mental health and job strain**

Word count: 4660 (excl, references, tables, and figures)

**Abstract**

**Objective:** The current study examined links between adverse mental health, job strain, and likelihood and frequency of primary care consultations among police employees. **Methods**: We conducted secondary data analysis on the Airwave Health Monitoring Study data (*n* = 33,730). Measures included self-report data on mental health, job strain, job support, and primary care consultations in the past 12 months. Data were analysed using a zero-inflated Poisson regression framework. **Results**: Findings showed that overall, help-seeking was low based on mental health status and job strain. Adverse mental health was associated with more primary care consultations. Police employees with high, active or passive job strain reported more primary care consultation compared to police employees with low strain. **Conclusions**: Findings suggest that more work to reduce stigma of taking the initial step of help-seeking would be beneficial.

**Keywords**: Police; help-seeking; primary care; mental health; job strain

**After reviewing this article, readers should be able to:**

- Identify the links between job strain, adverse mental health and primary care consultations
- Describe the impact of job support on help-seeking among police employees

**Primary care consultations among UK Police officers and staff: Links with adverse mental health and job strain**

Policing has consistently been linked with poor physical and mental health (1). Evidence has reported increased mental health problems among police employees, which is often attributed to occupational stressors (2). Police officers frequently rate occupational stressors, such as job strain, as more stressful than exposure to potentially traumatic events, such as dealing with physical aggression (3). Despite high rates of adverse mental health and occupational stressors, few police employees seek professional help (4). Indeed, symptoms of post-traumatic stress disorder (PTSD) have been linked to lower likelihood of organisational support service utilisation, such as workplace counselling, among police officers (5). However, little is known about how likely police employees are to utilise non-organisational health services, such as state-funded primary care health services. Understanding how likely police employees, at risk of adverse mental health, are to use primary care health services would allow employers to better cater for their health needs. The current study looked at whether adverse mental health, and high job strain are linked to both likelihood and frequency of primary care consultations among UK police employees.

The job demand-control model proposes that job strain depends on two key factors: job demands and job control (6). Job demands include aspects such as workload, time pressure, as well as physical and emotional demands. Job control refers to the extent individuals are capable of controlling their tasks and general work activity. According to the model, individuals in a high strain job (high demands and low control) are likely to report the lowest well-being (6). This model fits well with the results of a recent systematic review which identified job demand as being consistently linked with adverse mental health (7). Later iterations of the job demand-control model include perceived job support as a potential protective factor that might prevent negative outcomes otherwise associated with high job strain (8). Perceived job support includes aspects of social support at work (e.g., by colleagues or supervisors) and a supportive work environment (e.g., spirit of unity among employees). Indeed, recent guidelines from the world health organisation highlight the need to provide job support to effectively manage workers’ mental health (9). So far evidence for the protective effect of job support has been mixed with a systematic review of the model finding that only 4 out of 11 studies were supporting the protective effect of job support on general psychological well-being (10).

Findings among police employees have also been mixed. A recent study among Polish police officers supports the model, with high job support attenuating the association between high job demand and depressive symptoms (11). Similarly, among Norwegian police officers, both high job demand and low job support were associated with adverse mental health and burnout (12). In contrast, previous research among UK police employees from over 40,000 police employees in the Airwave study found no moderating effect of job support on the relationship between job strain and alcohol use or binge drinking (13). Thus, more research on the specific role of job strain on mental health status among police employees is needed. Further, it remains unknown whether police employees who experience high job strain are likely to seek professional help and whether job support increases such help-seeking. Conversely, it is also possible that high levels of job support may reduce the need for employees to seek help elsewhere.

While previous research on help-seeking by police employees has primarily focused on services offered by organisations (14), little information is available on the uptake of primary care consultations. This is important, as previous research indicates that police employees with mental health problems are more likely to seek help outside of their organisational support systems, in particular for those with problems relating to alcohol use and PTSD (15). Primary care physicians are often the first point of contact for individuals with mental health difficulties or those experiencing high levels of distress (16). Thus, understanding whether police employees with adverse mental health or high job strain (i.e., those likely in need of medical support) access primary care consultations and at what frequency is crucial in building better support systems. Despite provisions of organisational support services (e.g., workplace counselling), for the vast majority of workers, primary care services are the mainstay of health care, even when related to occupational matters (17). Effective organisational management of employee well-being is therefore reliant on both the provision of care and promotion of help-seeking more broadly, and requires knowledge about help-seeking within primary care.

To address these gaps, the current study aimed to examine the link between adverse mental health, job strain, and the likelihood and frequency of primary care consultations among police employees. Further, an interaction with job support was considered. To investigate the overlap between adverse mental health and job strain, we tested a potential interaction between these factors. Lastly, an exploratory analysis was conducted among “at-risk” police employees (i.e., probable mental disorder or high job strain) to examine which socio-demographic factors (e.g., age or sex) were associated with primary care consultations. an

**Methods**

**Study sample**

This study uses the Airwave Health Monitoring Study data (18). In brief, the Airwave study examined potential health impacts of the Terrestrial Trunked Radio (TETRA), a digital communication system used by emergency services since 2001. As part of the study police employees completed a self-report questionnaire and a health screen conducted by trained nurses. Data collection took place between June 2006 and March 2015. In total, 53,114 police employees participated from 28 participating forces across the UK, of whom 45,567 (85.8%) completed the self-report questionnaire and 45,514 (85.7%) completed the health screen. While no information is available on attrition, within most forces, both the percentage of men and the average age of participants were similar to those of the overall force (18). For this analysis, participants who indicated their role (i.e., police officer or police staff) as ‘other’ (*n* = 738) or did not have data available on their role (*n* = 8,245) were excluded due to limited confidence that responses were that of police employees. To minimise data reduction, we backfilled data on rank from employment data available through the Airwave study, which resulted in 94 completed cases. Data were used from all police employees who had answered the question regarding primary health care consultations as part of the self-report questionnaire (*n* = 33,730, 63.5% of the total sample). Ethical approval for The Airwave Health Monitoring Study was granted by the National Health Service multi-site research ethics committee (MREC/13/NW/0588). All participants provided written informed consent prior to participating.

**Measures**

**Primary care consultations***.* Participants were asked a single-item question on primary healthcare consultations in the previous year *(“In the past year how many times have you consulted your GP for your health problems?”*). Participants responded using a drop-down menu ranging from 0 to 20+ with each interval representing one consultation. Responses were treated as a count variable representing the number of primary care consultations in the previous year

**Adverse mental health***.* To examine adverse mental health, we used data on probable depression (Patient Health Questionnaire [PHQ-9];(19), probable anxiety disorder (Hospital Anxiety and Depression Scale – Anxiety [HADS-A]; (20), and probable post-traumatic stress disorder (PTSD) (Trauma Screening Questionnaire [TSQ]; (21). The PHQ-9 asked participants to indicate how frequently they had experienced nine different symptoms of depression over the previous two weeks on a 4-point Likert scale (0 = *not at all* to 3 *= nearly every day*). Probable depression was assigned using the validate cut-off score of 10 (19). Similarly, the HADS-A asked participants to rate the frequency of seven different symptoms of anxiety over the past two weeks on a 4-point Likert scale (0 = *not at all* to 3 *= most of the time/very much indeed*). Probable anxiety was assigned using the validated cut-off score of 11 (22). For the TSQ, participants were first asked whether they had been bothered by a disturbing incident that had occurred in the past six months. If they endorsed this item, participants were asked to indicate whether 10 different PTSD symptoms applied to them since that incident. Unlike the original TSQ measure, which used a yes/no response format, the Airwave Health Monitoring Survey asked participants to rate symptoms on a 5-point Likert scale (0 = *not at all* to 4 *= extremely*). Thus, in line with previous publications (Stevelink et al., 2020), all responses 1-4 were coded as 1. Probable PTSD was assigned using the validated cut-off score of 6 (21). All scales showed acceptable internal consistency (PHQ-9: α = .85; HADS-A: α = .67; TSQ: α = .92).

**Job demand, control and support.** As part of the Airwave Health Monitoring Survey, 10-items from the Job Content Questionnaire (JCQ; (23) were presented: Two items on job demands, four items on job control, and four items on support. Participants rated how much they agreed with each item on a 4-point Likert scale (1 = *strongly agree/often* to 4 *= strongly disagree/never*). Based on the original scale model, participants were categorised into four distinct categories of job strain using a quadrant approach based on the sample median: *high strain* (high demands, low control), *low strain* (low demands, high control), *active strain* (high demands, high control), and *passive strain* (low demands, low control). Job support was included as a separate continuous variable, with scores ranging from 4-16 whereby higher scores indicate higher perceived job support. All subscales demonstrated acceptable internal consistency (job demand: α = .73; job control: α = .67; job support: α = .83).

**Control variables**

**Socio-demographic***.* To control for socio-demographic influences on primary care consultations, we included the following factors as control variables: Rank (police officer or police staff), years in current role, salary, educational attainment, marital status, ethnicity, age and sex.

**Job Satisfaction.** Participants were asked to rate their job satisfaction on a single item using a 4-point Likert scale (1 = *very dissatisfied* to 4 *= very satisfied).* Scores on this variable were highly skewed, with most participants rating their job satisfaction as either satisfied or very satisfied (82.29%), and assumption of a normal distribution was violated (Shapiro Wilk test: *z* = 11.74, *p* < .001). As such, this variable was treated as a categorical variable with two levels: *very dissatisfied/dissatisfied* and *very satisfied/satisfied*.

**Physical health***.* To control for participant’s physical health, we included the following factors as control variables: Self-reported health condition (yes/no on any health condition excluding depression), and diastolic blood pressure (taken by a nurse at a separate health assessment). In the list of health conditions, depression was the only listed mental health problem. As the purpose of controlling for physical health indicators was to minimise the influence of physical health factors on help-seeking, we did not include self-reported depression in this variable. As some participants presented to the health assessment multiple times, we excluded data on blood pressure from these participants, as it was unclear which visit corresponded with their survey data. In line with NHS guidelines, we categorised diastolic blood pressure into three categories: low diastolic blood pressure (<60mmHg), normal diastolic blood pressure (60-89mmHg), and high diastolic blood pressure (90mmHg or higher).

**Data analysis plan**

The data analysis plan was pre-registered (https://osf.io/awgme/?view_only=e4d0c2bad44a4496b3bd88ab2a188ced). All deviations from this plan are explicitly mentioned below. As planned, analyses were conducted in a zero-inflated Poisson regression framework in Stata version 16 (24). The zero-inflated Poisson regression uses a mixture of a Poisson distribution of count data (e.g., number of primary care consultations) with an excess of zero counts. Using this regression, the occurrence of the outcome (zero-inflated part) and the frequency of the outcome (Poisson part) were examined separately in the same model.

To test the study aims we conducted four separate regression analyses. The main outcome of interest was two-fold: 1) likelihood of at least one past year primary care consultations and 2) frequency of past year primary care consultations. The first model examined the link between adverse mental health and primary care consultations likelihood and frequency, the second model examined the link between job strain and primary care consultations, and finally, the third model included both factors (adverse mental health and job strain). In the final model, we also tested the interaction between these factors. For simplicity, we used a combined “any mental health problem” to capture individuals who met criteria for either a probable anxiety disorder, depression or PTSD in the final model. The first two models additionally considered an interaction with job support. We had originally planned to include binge drinking as another facet, alongside adverse mental health and job strain. However, these analyses will be reported in a future publication instead.

All analyses controlled for socio-demographic factors as well as physical health factors, that had a significant association with primary care consultations in univariate analyses. To deal with missing data, we first examine missing data patterns to determine whether data were missing at random. In total, there were 8% of missing data. Missing data were significantly associated with sex, job rank, salary, age, and education. To account for these associations, these factors were controlled for in all analyses, even if no significant associations with primary care consultations emerged in univariate analyses. While we had initially planned to use multiple imputation to deal with missing data, this was not deemed appropriate given the data were not missing at random (25). Instead, we used list-wise deletion to deal with missing data (26,27).

For exploratory analyses, we examined socio-demographic factors linked with primary care consultations for individuals who met the criteria for “any mental health problem” or were classified as experiencing ‘high job strain’. Analyses were carried out in a zero-inflated Poisson regression. Physical health variables were included as covariates.

**Results**

**Sample characteristics**

The sample characteristics (*n* = 33,730) are described in Table 1. Both the ethnicity and sex composition of the sample is representative of the broader police force, where 92.4% of police officers are White and 67.6% are male (28).

In total, 38.6% of the sample reported at least one health condition out of a list of 20 conditions, excluding mental health problems. Of these, the most common health conditions were allergies (*n* = 5,187, 39.8%), asthma (*n* = 3,408, 26.2%), and migraine (*n* = 2,536, 19.5%). Most participants with a health condition reported having one (70.7%) or two conditions (23.3%). In terms of primary care consultations, most people reported having at least one primary care consultation in the past 12 months (n = 23,342, 69.2%), with a mean of 1.7 consultations (*SD* = 2.1; Mdn =1; IQR = 0-2). Primary care consultations in the current sample were substantially lower compared to the average UK population (i.e., including non-working individuals), with an average of 3.8 primary care consultations over 12 months (29).

[insert Table 1 here]

**Univariable associations**

Table 1 shows findings from the univariate analyses examining the association between socio-demographic and occupational characteristics, and physical health with both the likelihood of attending a primary care consultation and the frequency of primary care consultations in the previous 12 months. Police employees were *more likely to attend a primary care consultation within the past 12 month* if they were under 30 years of age, female, non-White ethnicity, police staff, in their current role for less than five years, had a lower salary, worked fewer hours, had low blood pressure, and reported a medical condition. Police employees *attended more primary care consultations in the past 12 months* if they were aged over 50, female, non-White ethnicity, did not complete their A levels, were not married or cohabitating, police staff, in their current role for 11-20 years, had a lower salary, worked fewer hours, had high blood pressure, and reported a medical condition.

Based on these findings, all socio-demographic, occupational and physical health variables were included as covariates in the subsequent multivariate analyses.

**Main analyses**

**Adverse mental health.** A total of 6,074 (19.2%) participants reported meeting criteria for a mental health problem. In total, 9.8% met the criteria for probable depression, 12.2% for probable anxiety and 4.0% for probable PTSD. Findings showed that only police employees with a probable anxiety disorder were more likely to have attended a primary care consultation in the past 12 months, compared to police employees without an anxiety disorder (see Table 2). However, police employees with probable depression, anxiety, and/or PTSD had more primary health consultations in the past 12 months compared to police employees without mental health problems. Notably, this association was stronger for probable depression compared to probable anxiety disorder or probable PTSD.

[insert Table 2 here]

Additionally, there was an interaction between job support and probable PTSD, but not probable depression or anxiety, in the association with frequency of primary care consultations. As can be seen in Figure 1, higher levels of job support were associated with more primary care consultations among police employees with probable PTSD, but not among those without probable PTSD.

[insert Figure 1 here]

**Job strain.** Around 27.7% of participants were categorised as having low job strain, 23.7% as high job strain, 28.3% as active job strain and 20.3% were categorised as having passive job strain. Police employees with high strain, active strain or passive strain attended more primary care consultations than police employees with low strain (see Table 3). This effect was most pronounced for those in the high strain category. Job support was not associated with likelihood or frequency of primary care consultations, nor did it interact with job strain.

[insert Table 3 here]

**Combined factors.** When examining adverse mental health and job strain together, findings remained largely unchanged (see Table 4). One exception was passive job strain, which was no longer associated with frequency of primary care consultation when compared to low strain.

Further, there was a significant interaction between adverse mental health and high job strain. Specifically, among police employees with a probable mental health problem, there was no difference in primary care consultations between police employees experiencing high job strain compared to those experiencing low job strain. In contrast, among police employees without a mental health problem, those with high job strain had more primary care consultations compared to those with low job strain. Additionally, there was an interaction between adverse mental health and passive job strain. That is, police employees with a probable mental health problem with passive job strain were more likely to attend a primary care consultation compared to those without a mental health problem and low job strain.

[insert Table 4 here]

**Exploratory analysis**

As exploratory analyses, we examined the socio-demographic factors linked with primary care consultations among police officers with a probable mental health problem or those experiencing high job strain (*n* = 11,461; 34.3%) – that is, those deemed “at risk” for psychological problems requiring formal health care (see Table S1, <http://links.lww.com/JOM/B304>). Few significant associations emerged when examining likelihood of primary care consultations. Police employees deemed to be “high-risk” were more likely to attend a primary care consultation if they were female, married or cohabitating as opposed to single, police staff, or in their current role for less than 5 years. Several factors were linked to frequency of primary care consultations. Police employees deemed to be “high-risk” had attended more primary care consultations if they were older, female, non-White, were not married, had a lower salary, and had fewer working hours. The only factor associated with both reduced likelihood and frequency of primary care consultations was being male, which also had the biggest effect size. Conversely, being single compared to married/cohabitating was associated with lower likelihood of attending a primary care consultation, but higher frequency.

**Discussion**

The current study aimed to examine the link between adverse mental health, job strain, and the likelihood and frequency of primary care consultations among UK police employees. Findings showed that police employees with a probable anxiety disorder were more likely to attend a primary care consultation, compared to those without a probable anxiety disorder. Additionally, adverse mental health was associated with frequency of primary care consultations, particularly for those with probable depression. Neither probable depression nor probable PTSD had a unique association with higher likelihood of primary care consultations. Thus, there may be a difference between different types of adverse mental health and likelihood of attending primary care consultations that should be investigated further. Overall, adverse mental health reported by police employees was similar to that in the general working population (30), and similar occupation groups like the military (31,32).

When examining job strain, findings were in line with the job demand-control model (6), whereby effects were most pronounced for police employees reporting high job strain. That is, police employees with high job strain, active job strain, and passive job strain reported more frequent primary care consultations compared to police employees with low strain. There was no difference in likelihood of primary care consultations. Consequently, police employees with higher job strain may not necessarily recognise the need to seek help, but do require more medical attention. This is in line with previous research among the general working population, where links between job strain and sick days have frequently been reported (33,34). Thus, this study provides further evidence for the link between job strain and health problems. However, job support appeared to have no notable links with primary care consultations in the current study. Interestingly, there was a significant interaction between job support and probable PTSD in that higher job support was associated with more primary care consultations among police employees with probable PTSD, but not those without. These findings suggest that while job support has little influence on help-seeking among police employees, job support can be beneficial for those most in need of additional help. Further, as police work is inherently linked to potentially traumatic exposures, PTSD may be more recognised by managers and spoken about in a work setting, which may foster a supportive environment that facilitates help-seeking.

Further, the current study found a significant interaction between adverse mental health and job strain. That is, among police employees without a mental health problem, those with high job strain had more primary care consultations compared to those with low job strain. In contrast, among police employees who reported a probable mental health problem, those experiencing high job strain did not differ in primary care consultations compared to those experiencing low job strain. This suggests that while police employees without a probable mental health problem might be more likely to notice the additional support needed to handle the impact of experiencing high job strain, those also experiencing adverse mental health may be less likely to recognise the additional impact of high job strain on their mental health. Indeed, previous research has found that one of the mechanisms linking high job strain with adverse mental health is a lack of detachment (i.e., thinking about work when at home; (35). Police employees with adverse mental health may already experience problems with detachment, regardless of job strain, unlike police employees without adverse mental health for whom the impact of high job strain may be more noticeable.

Findings from our exploratory analyses found few structural factors related to likelihood of primary care consultations among those “at risk”. A notable finding was the strong association between sex and both likelihood and frequency of primary care consultations, with men significantly less likely to attend a primary care consultation and attending fewer consultations compared to women. While this is in line with more general sex differences in mental health help-seeking (36), it provides an important avenue for the police to actively encourage male employees to seek help and tackle both stigma towards mental health in general, but also gender-based stigma.

The current study has several implications for organisational support within the police force and similar organisational structures. Firstly, the current study suggests that police employees who might benefit from formal medical support (i.e., those with adverse mental health, or high job strain) report the same likelihood of attending primary care consultations compared to their peers. However, there was evidence that police employees with adverse mental health or high job strain attended more primary care consultations. Thus, efforts should focus on encouraging police employees to take the initial step of seeing their primary care physician, as current findings suggest that once police employees have engaged with primary care, they will continue with treatment as needed. Stigma is a frequently reported barrier for police employees seeking help for mental health problems (5), which many police employees experience as a key part of police culture (37). This may be addressed through organisational efforts, although it is important to note that stigma is a societal issue not restricted to the police force (38). Indeed, a recent study among Canadian police employees found that having completed mental health training (i.e., Mental Health First Aid course) was positively associated with help-seeking (39). Additionally, the current study highlights the impact of job strain on mental health, with police employees experiencing high, active, or passive job strain reporting significantly more primary care consultations compared to police employees with low job strain. While it is encouraging to see this level of help-seeking, it further reinforces the adverse impact of low job control and high job demands. Thus, police forces should strive to further reduce these factors to promote well-being among employees.

While the current study had many strengths, including the large representative sample, several limitations should be noted. Firstly, the study relied purely on self-report data. In particular, reporting on primary care consultations in the previous 12 months may have been subject to recall bias with police employees potentially under or overestimating the number of consultations they attended. Specifically, recall bias may differ between individuals with a probable mental health problem and those without a mental health problem, whereby individuals with mental health problems frequently overestimate negative events and underestimate positive events (40). Secondly, the data were collected over several years during which several changes in health care were implemented. For example, in 2008 the UK introduced an alternative pathway to accessing low-intensity mental health treatment (Improving Access to Psychological Therapies [IAPT]), which people can access without a referral from their primary care physician (41) and is commonly used for treating low-level depression and anxiety. Thus, police employees may have visited their primary care physician less, but attended IAPT sessions instead. However, no information was available on this in the current study. Similarly, the study did not examine the reasons for a primary care consultation, and we do not know whether police employees were attending consultations to seek help for their mental health. While we did control for some aspects of physical health (e.g., blood pressure and history of a medical condition), future research should examine mental health help-seeking more explicitly. Additionally, primary care consultations referred to the 12 past months only and we did not have historical data available. It is therefore unknown to what degree participants accessed primary care consultations in the preceding years. Lastly, the current study relied on cross-sectional data and no inferences about causality can be made. Future research should examine whether experiences of adverse mental health and high job strain lead to subsequent primary care consultations.

In conclusion, the current study showed that police employees with adverse mental health and high job strain reported more primary care consultations in the previous 12 months compared to police employees without adverse mental health and low job strain. However, while some police employees with adverse mental health were also more likely to have had any primary care consultations, this association was not observed for police employees with high job strain. These results suggest that more work to reduce stigma of taking the initial step of help-seeking would be beneficial. This is especially true for male police staff who were the least likely to seek help from primary care. Furthermore, whilst it is laudable that high job support was linked with more frequent primary care consultations among police employees with probable PTSD, it would be useful to help police managers better understand the potential benefits of staff with anxiety disorders or depression also seeking help, especially if their symptoms are severe and impairing occupational function.

**References**

1. Violanti JM, Charles LE, McCanlies E, Hartley TA, Baughman P, Andrew ME, et al. Police stressors and health: a state-of-the-art review. Policing [Internet]. 2017;40(4):642–56. Available from: www.emeraldgrouppublishing.com/licensing/

2. Syed S, Ashwick R, Schlosser M, Jones R, Rowe S, Billings J. Global prevalence and risk factors for mental health problems in police personnel: A systematic review and meta-analysis [Internet]. Vol. 77, Occupational and Environmental Medicine. 2020. p. 737–47. Available from: http://dx.doi.org/10.1136/oemed-2020-106498

3. Collins PA, Gibbs ACC. Stress in police officers: A study of the origins, prevalence and severity of stress-related symptoms within a county police force. Occup Med (Chic Ill) [Internet]. 2003;53(4):256–64. Available from: https://academic.oup.com/occmed/article/53/4/256/1442925

4. Berg AM, Hem E, Lau B, Ekeberg Ø. Help-seeking in the Norwegian police service. J Occup Health [Internet]. 2006 May 1 [cited 2022 Jul 27];48(3):145–53. Available from: https://onlinelibrary.wiley.com/doi/full/10.1539/joh.48.145

5. Ménard KS, Arter ML, Khan C. Critical incidents, alcohol and trauma problems, and service utilization among police officers from five countries. Int J Comp Appl Crim Justice [Internet]. 2016 Jan 2;40(1):25–42. Available from: https://www.tandfonline.com/action/journalInformation?journalCode=rcac20

6. Karasek R. Job Demands, Job Decision Latitude, and Mental Strain: Implications for Job Redesign. Adm Sci Q [Internet]. 1979 Aug 26;24(2):285–308. Available from: http://www.jstor.org/stable/2392498

7. Purba A, Demou E. The relationship between organisational stressors and mental wellbeing within police officers: A systematic review. BMC Public Health [Internet]. 2019;19(1). Available from: https://doi.org/10.1186/s12889-019-7609-0

8. Johnson J v, Hall EM. Job strain, work place social support, and cardiovascular disease: a cross-sectional study of a random sample of the Swedish working population. Am J Public Health [Internet]. 1988 Oct 1;78(10):1336–42. Available from: https://doi.org/10.2105/AJPH.78.10.1336

9. World Health Organization. WHO guidelines on mental health at work. 2022.

10. Häusser JA, Mojzisch A, Niesel M, Schulz-Hardt S. Ten years on: A review of recent research on the Job Demand-Control (-Support) model and psychological well-being. Work Stress [Internet]. 2010 Jan;24(1):1–35. Available from: http://www.tandfonline.com/doi/abs/10.1080/02678371003683747

11. Baka L. Types of job demands make a difference. Testing the job demand-control-support model among Polish police officers. International Journal of Human Resource Management [Internet]. 2020 Oct 10 [cited 2022 Aug 10];31(18):2265–88. Available from: https://www.tandfonline.com/doi/abs/10.1080/09585192.2018.1443962

12. Berg AM, Hem E, Lau B, Ekeberg Ø. An exploration of job stress and health in the Norwegian police service: A cross sectional study. Journal of Occupational Medicine and Toxicology [Internet]. 2006 Dec 11 [cited 2022 Aug 10];1(1):1–9. Available from: https://occup-med.biomedcentral.com/articles/10.1186/1745-6673-1-26

13. Irizar P, Gage SH, Field M, Fallon V, Goodwin L. The prevalence of hazardous and harmful drinking in the UK Police Service, and their co-occurrence with job strain and mental health problems. Epidemiol Psychiatr Sci [Internet]. 2021 Jun 21;30:e51. Available from: https://www.cambridge.org/core/product/identifier/S2045796021000366/type/journal_article

14. Kleim B, Westphal M. Mental Health in First Responders: A Review and Recommendation for Prevention and Intervention Strategies. Traumatology (Tallahass Fla) [Internet]. 2011;17(4):17–24. Available from: http://tmt.sagepub.com

15. Fox J, Desai MM, Britten K, Lucas G, Luneau R, Rosenthal MS. Mental-health conditions, barriers to care, and productivity loss among officers in an urban police department. Conn Med [Internet]. 2012 Oct [cited 2022 Aug 10];76(9):525–31. Available from: /pmc/articles/PMC4089972/

16. Kovess-Masfety V, Alonso J, Brugha TS, Angermeyer MC, Haro JM, Sevilla-Dedieu C, et al. Differences in lifetime use of services for mental health problems in six European countries. Psychiatric Services. 2007;58(2):213–20.

17. Hussey L, Turner S, Thorley K, McNamee R, Agius R. Work-related ill health in general practice, as reported to a UK-wide surveillance scheme. British Journal of General Practice. 2008;58(554):637–40.

18. Elliott P, Vergnaud AC, Singh D, Neasham D, Spear J, Heard A. The airwave health monitoring study of police officers and staff in great britain: Rationale, design and methods. Environ Res. 2014 Oct 1;134:280–5.

19. Kroenke K, Spitzer RL, Williams JBW. The PHQ-9: Validity of a brief depression severity measure. J Gen Intern Med. 2001;16(9):606–13.

20. Zigmond AS, Snaith RP. The Hospital Anxiety and Depression Scale. Acta Psychiatr Scand [Internet]. 1983 [cited 2022 Jul 25];67(6):361–70. Available from: https://pubmed.ncbi.nlm.nih.gov/6880820/

21. Brewin CR, Rose S, Andrews B, Green J, Tata P, McEvedy C, et al. Brief screening instrument for post-traumatic stress disorder. British Journal of Psychiatry [Internet]. 2002;181(2):158–62. Available from: https://doi.org/10.1192/bjp.181.2.158

22. Bjelland I, Dahl AA, Haug TT, Neckelmann D. The validity of the Hospital Anxiety and Depression Scale. J Psychosom Res [Internet]. 2002 Feb 1 [cited 2022 Jul 25];52(2):69–77. Available from: https://linkinghub.elsevier.com/retrieve/pii/S0022399901002963

23. Karasek R, Brisson C, Kawakami N, Houtman I, Bongers P, Amick B. The Job Content Questionnaire (JCQ): an instrument for internationally comparative assessments of psychosocial job characteristics. J Occup Health Psychol. 1998 Oct;3(4):322–55.

24. StataCorp. Stata Statistical Software: Release 16. College Station, TX: StataCorp LLC; 2019.

25. Sterne JAC, White IR, Carlin JB, Spratt M, Royston P, Kenward MG, et al. Multiple imputation for missing data in epidemiological and clinical research: potential and pitfalls. BMJ [Internet]. 2009 Sep 1;338(jun29 1):b2393–b2393. Available from: http://www.bmj.com/content/338/bmj.b2393.abstract

26. Pepinsky TB. A Note on Listwise Deletion versus Multiple Imputation. Political Analysis [Internet]. 2018;26(4):480–8. Available from: https://doi.org/10.1017/pan.2018.18

27. Hughes RA, Heron J, Sterne JAC, Tilling K. Accounting for missing data in statistical analyses: Multiple imputation is not always the answer. Int J Epidemiol. 2019;48(4):1294–304.

28. Harding M. Police Service Strength [Internet]. 2021. Available from: https://researchbriefings.files.parliament.uk/documents/SN00634/SN00634.pdf

29. Hobbs FDR, Bankhead C, Mukhtar T, Stevens S, Perera-Salazar R, Holt T, et al. Clinical workload in UK primary care: a retrospective analysis of 100 million consultations in England, 2007–14. The Lancet [Internet]. 2016 Jun 4 [cited 2022 Sep 7];387(10035):2323–30. Available from: http://www.thelancet.com/article/S0140673616006206/fulltext

30. McManus S, Bebbington P, Jenkins R, Brugha T. Mental health and wellbeing in England: Adult Psychiatric Morbidity Survey 2014. NHS Digital. Leeds; 2016.

31. Irizar P, Stevelink SAM, Pernet D, Gage SH, Greenberg N, Wessely S, et al. Probable post-traumatic stress disorder and harmful alcohol use among male members of the British Police Forces and the British Armed Forces: a comparative study. Eur J Psychotraumatol [Internet]. 2021;12(1). Available from: https://doi.org/10.1080/20008198.2021.1891734

32. Stevelink SAM, Jones M, Hull L, Pernet D, Maccrimmon S, Goodwin L, et al. Mental health outcomes at the end of the British involvement in the Iraq and Afghanistan conflicts: A cohort study. British Journal of Psychiatry. 2018;213(6):690–7.

33. Mather L, Bergström G, Blom V, Svedberg P. High job demands, job strain, and iso-strain are risk factors for sick leave due to mental disorders a prospective Swedish twin study with a 5-year follow-up. J Occup Environ Med [Internet]. 2015 Sep 1 [cited 2022 Jul 29];57(8):858–65. Available from: https://journals.lww.com/joem/Fulltext/2015/08000/High_Job_Demands,_Job_Strain,_and_Iso_Strain_Are.5.aspx

34. Virtanen M, Vahtera J, Pentti J, Honkonen T, Elovainio M, Kivimäki M. Job Strain and Psychologic Distress. Influence on Sickness Absence Among Finnish Employees. Am J Prev Med. 2007 Sep 1;33(3):182–7.

35. Matick E, Kottwitz MU, Lemmer G, Otto K. How to sleep well in times of high job demands: The supportive role of detachment and perceived social support. Work Stress [Internet]. 2021;35(4):358–73. Available from: https://doi.org/10.1080/02678373.2021.1889071

36. Pattyn E, Verhaeghe M, Bracke P. The gender gap in mental health service use. Soc Psychiatry Psychiatr Epidemiol. 2015;50(7):1089–95.

37. Edwards AM, Kotera Y. Mental Health in the UK Police Force: a Qualitative Investigation into the Stigma with Mental Illness. Int J Ment Health Addict [Internet]. 2021 Aug 1 [cited 2022 Sep 7];19(4):1116–34. Available from: https://link.springer.com/article/10.1007/s11469-019-00214-x

38. Woodhead C, Rona RJ, Iversen A, MacManus D, Hotopf M, Dean K, et al. Mental health and health service use among post-national service veterans: results from the 2007 Adult Psychiatric Morbidity Survey of England. Psychol Med [Internet]. 2010/04/21. 2011;41(2):363–72. Available from: https://www.cambridge.org/core/article/mental-health-and-health-service-use-among-postnational-service-veterans-results-from-the-2007-adult-psychiatric-morbidity-survey-of-england/B313DD58546F643009E5E9F5C3DAAD47

39. Lane J, Le M, Martin K, Bickle K, Campbell E, Ricciardelli R. Police Attitudes Toward Seeking Professional Mental Health Treatment. J Police Crim Psychol [Internet]. 2022;37(1):123–31. Available from: https://doi.org/10.1007/s11896-021-09467-6

40. Colombo D, Suso-Ribera C, Fernández-Álvarez J, Cipresso P, Garcia-Palacios A, Riva G, et al. Affect Recall Bias: Being Resilient by Distorting Reality. Cognit Ther Res [Internet]. 2020;44(5):906–18. Available from: https://doi.org/10.1007/s10608-020-10122-3

41. NHS England. NHS England » IAPT at 10: Achievements and challenges [Internet]. 2019 [cited 2022 Sep 20]. Available from: https://www.england.nhs.uk/blog/iapt-at-10-achievements-and-challenges/

Figure legend:

*Figure 1. Interaction between job support and probable PTSD regarding their association with frequency of primary care consultations.*
