# Supplementary material for: Primary Care Consultations Among UK Police Officers and Staff: Links With Adverse Mental Health and Job Strain
Source: J Occup Environ Med. 2023 Feb 26;65(6):502–9. doi: 10.1097/JOM.0000000000002819 (PMC10227927; doi:10.1097/JOM.0000000000002819)
Supplement: Supplementary file 2 [file joem-65-0502-s002.docx]

**Primary care consultations among UK Police officers and staff: Links with adverse mental health and job strain**

Supplementary materials

**Table S1.** Regression analysis examining likelihood and frequency of primary care consultations by socio-demographic factors among “high risk” police employees (i.e., probable mental health condition, or high job strain)

| Primary care consultations | Likelihood | | | Frequency | | |  |  |
| --- | --- | --- | --- | --- | --- | --- | --- | --- |
|  | AOR | *p*-value | 95% CI | IRR | *p*-value | 95% CI | |  |
| Age (in years) |  |  |  |  |  |  | |  |
| <30 | reference | | | | | | |  |
| 30-39 | 0.87 | .251 | [0.69, 1.10] | **1.15** | **<.001** | **[1.09, 1.20]** | |  |
| 40-49 | 0.88 | .292 | [0.68, 1.12] | **1.18** | **<.001** | **[1.12, 1.24]** | |  |
| ≥50 | 0.95 | .738 | [0.69, 1.29] | **1.23** | **<.001** | **[1.16, 1.31]** | |  |
| Sex |  |  |  |  |  |  | |  |
| Female | Reference | | | | | | |  |
| Male | **0.64** | **<.001** | **[0.55, 0.76]** | **0.74** | **<.001** | **[0.71, 0.76]** | |  |
| Ethnicity |  |  |  |  |  |  | |  |
| White | Reference | | | | | | |  |
| Non-White | 1.31 | .100 | [0.95, 1.80] | **1.14** | **<.001** | **[1.08, 1.21]** | |  |
| Education |  |  |  |  |  |  | |  |
| Low (O levels/GCSEs or none) | Reference | | | | | | |  |
| High (A levels, degree or higher) | .99 | .936 | [0.85, 1.16] | 0.98 | .190 | [0.95, 1.01] | |  |
| Marital status |  |  |  |  |  |  | |  |
| Married/cohabiting | Reference | | | | | | |  |
| Divorced/separated | 1.01 | .938 | [0.79, 1.29] | **1.09** | **.001** | **[1.03, 1.14]** | |  |
| Single | **0.75** | **.004** | **[0.62, 0.91]** | **1.07** | **.001** | **[1.03, 1.12]** | |  |
| Other | 0.86 | .498 | [0.57, 1.32] | 1.09 | .049 | [1.00, 1.20] | |  |
| Rank |  |  |  |  |  |  | |  |
| Police officer | Reference | | | | | | |  |
| Police staff | **1.42** | **.001** | **[1.15, 1.76]** | 0.99 | .756 | [0.95, 1.04] | |  |
| Years in current role |  |  |  |  |  |  | |  |
| ≤ 5 years | Reference | | | | | | |  |
| 6-10 years | 0.88 | .113 | [0.73, 1.04] | 0.97 | .144 | [0.94, 1.01] | |  |
| 11-20 years | **0.77** | **.021** | **[0.62, 0.96]** | 1.02 | .504 | [0.97, 1.07] | |  |
| ≥ 20 years | 1.13 | .593 | [0.71, 1.80] | 0.93 | .113 | [0.85, 1.02] | |  |
| Salary |  |  |  |  |  |  | |  |
| ≤ £25999 | Reference | | | | | | |  |
| £26000 - £37999 | 1.02 | .852 | [0.83, 1.26] | 1.02 | .347 | [0.98, 1.06] | |  |
| £38000 – £59999 | 1.11 | .439 | [0.86, 1.42] | **0.89** | **<.001** | **[0.84, 0.93]** | |  |
| ≥ £60000 | 0.90 | .744 | [0.49, 1.67] | **0.76** | **<.001** | **[0.65, 0.88]** | |  |
| Total working hours (excluding overtime) |  |  |  |  |  |  | |  |
| ≤40 hours per week | Reference | | | | | | |  |
| 41-48 hours per week | 0.99 | .892 | [0.80, 1.22] | **0.93** | **<.001** | **[0.89, 0.97]** | |  |
| ≥49 hours per week | 1.00 | .969 | [0.75, 1.31] | **0.90** | **<.001** | **[0.85, 0.95]** | |  |

*Note.* Significant associations are bolded. Analysis adjusted for blood pressure and medical diagnosis. AOR = Adjusted odds ratio. IRR = Incidence rate ratio.
